# ID1 and IGFBP3: roles in cellular senescence, cardiac development, angiogenesis and cancer diagnosis

**DOI:** 10.1186/s12967-023-04701-7

**Published:** 2023-11-09

**Authors:** Cheng Shen

**Affiliations:** Department of Thoracic Surgery, West-China Hospital, Sichuan University, Chengdu, 610041 China

## To the editor,

**Fig. 1:**
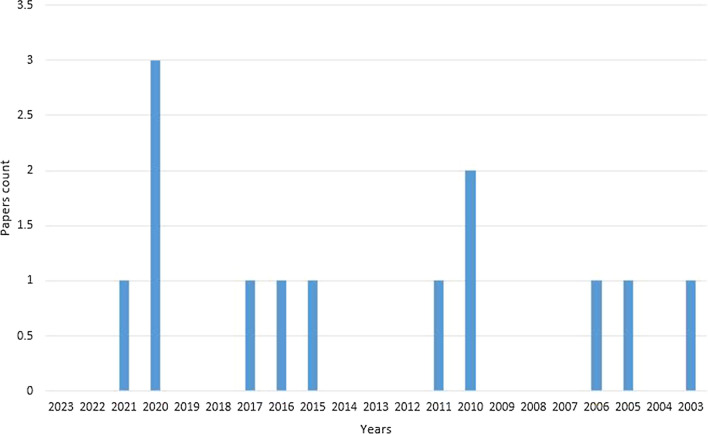
Number of publications of ID1 and IGFBP3 in PubMed

**Fig. 2:**
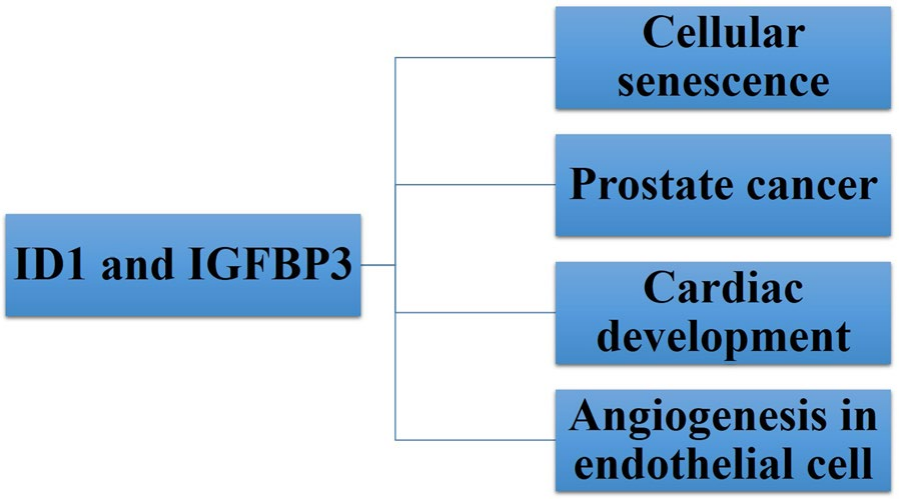
Relative contents on the role of ID1 and IGFBP3

The protein known as Inhibitor of DNA-binding 1 (ID1) is a component of the helix–loop–helix (HLH) group of proteins [1]. Insulin-like growth factor binding protein-3 (IGFBP3) exerts its influence by managing the bioavailability of the insulin-like growth factor (IGF) [2]. After a complete search for infinite definite words of “ID1 and IGFBP3” in PubMed, only 12 references were found from 2003 to 2023 years (Fig. 1) and we summarized the relative literatures on the relationship between ID1 and IGFBP3 (Fig. 2).

## ID1 and IGFBP3 in cellular senescence

TGF-β1 is postulated to be a primary factor in prostate aging, facilitating premature senescence and promoting myofibroblast differentiation [3]. Both cellular senescence and TGF-β-induced growth arrest and differentiation of prostate basal cells lead to the downregulation of ID1. Furthermore, IGFBP3 mRNA levels are reduced after a 24-h exposure to TGF-β1, 2 and 3 [3].

## ID1 and IGFBP3 in prostate cancer

Research indicates that normal prostate epithelial cells display low or virtually non-existent ID1 expression levels, contrasting with the heightened levels observed in prostate cancer. Therefore, the integration of ID1 into normal prostate epithelial cells might present a method for examining the early stages involved in the onset of prostate cancer. IGFBP3 has been discovered to exhibit both inhibitory and stimulatory effects on cells, with these effects being mediated via specific IGFBP3 binding proteins/receptors [1].

## ID1 and IGFBP3 in cardiac development

Early research reported that ID1 and ID3 double knockout (dKO) mouse embryos suffer mid-gestation demise due to multiple cardiac defects [4]. Changes in the expression patterns of vascular, fibrotic, and hypertrophic markers were observed in the ID dKO hearts, but IGFBP3 introduction restored vascular and fibrotic gene expression patterns [4]. This implies that deletion of ID genes in the vasculature results in distinct postnatal cardiac phenotypes and highlight IGFBP3 as a possible link between ID and its vascular effectors [4]. It is plausible that ID1 suppresses IGFBP3 within the endothelium, given that both proteins have been found there.

## ID1 and IGFBP3 in angiogenesis in endothelial cell

Bone morphogenetic protein 2 (BMP2) is crucial for endometrial decidualization and the invasion of trophoblast cells [2, 5]. Interestingly, IGFBP3 promotes cell migration and angiogenesis in endothelial cells, while ID1 has a regulatory impact on IGFBP3 expression in rat prostate epithelial cells [2]. The researchers verified that ID1 and IGFBP3 promote human trophoblast invasion and the formation of endothelial-like tubes. Additionally, they showed that ID1 plays a role in mediating the BMP2-induced increase in IGFBP3 expression [5].

In conclusion, ID1 and IGFBP3 each have their own unique biological properties, and there are currently few research results on the relationship between them. Hence, exploring the mechanisms that underlie this specificity would be a compelling area for future research.

## Data Availability

All data for this study are publicly available and are ready for the public from database of hospital.

## References

[1] Schmidt M, Asirvatham AJ, Chaudhary J (2010). Inhibitor of differentiation 1 (ID1) promotes cell survival and proliferation of prostate epithelial cells. Cell Mol Biol Lett.

[2] Luo J, Zhu H, Chang HM, Lin YM, Yang J, Leung PCK (2020). The regulation of IGFBP3 by BMP2 has a role in human endometrial remodeling. FASEB J.

[3] Untergasser G, Gander R, Rumpold H, Heinrich E, Plas E, Berger P (2003). TGF-beta cytokines increase senescence-associated beta-galactosidase activity in human prostate basal cells by supporting differentiation processes, but not cellular senescence. Exp Gerontol.

[4] Chang C, Zhao Q, Gonzalez JP (2017). Hematopoietic Id deletion triggers endomyocardial fibrotic and vascular defects in the adult heart. Sci Rep.

[5] Yi Y, Zhu H, Klausen C (2021). Dysregulated BMP2 in the placenta may contribute to early-onset preeclampsia by regulating human trophoblast expression of extracellular matrix and adhesion molecules. Front Cell Dev Biol.

